# β-adrenoceptor activation increased VAMP-2 and syntaxin-4 in secretory granules are involved in protein secretion of submandibular gland through the PKA/F-actin pathway

**DOI:** 10.1042/BSR20171142

**Published:** 2018-02-13

**Authors:** Chong Ding, Xin Cong, Yan Zhang, Sheng-Lin Li, Li-Ling Wu, Guang-Yan Yu

**Affiliations:** 1 Center Laboratory, Peking University School and Hospital of Stomatology, National Engineering Laboratory for Digital and Material Technology of Stomatology and Beijing Key Laboratory of Digital Stomatology, Beijing, China; 2 Department of Physiology and Pathophysiology, Peking University School of Basic Medical Sciences, Key Laboratory of Molecular Cardiovascular Sciences, Ministry of Education and Beijing Key Laboratory of Cardiovascular Receptors Research, Beijing, China; 3 Department of Oral and Maxillofacial Surgery, Peking University School and Hospital of Stomatology, Beijing, China

**Keywords:** β-adrenoceptors, F-actin, protein kinase A, synaptobrevin/vesicle-associated membrane protein-2, syntaxin-4

## Abstract

Autologous submandibular gland transplantation is an effective treatment for severe dry eye syndrome. However, the protein secretion in transplanted gland is altered by a mechanism that remains to be elucidated. In the present study, we found that β1-adrenoceptor (β1-AR) and β2-AR expression and the phosphorylation of the downstream molecule protein kinase A (PKA) were elevated in transplanted submandibular glands obtained from epiphora patients. Synaptobrevin/vesicle-associated membrane protein 2 (VAMP-2) interacted with syntaxin-4 and actin in human submandibular gland. The contents of syntaxin-4 and actin interacting with VAMP-2 were increased in transplanted gland. Moreover, VAMP-2 and syntaxin-4 expression in the secretory granule fraction, and VAMP-2 expression in the membrane protein fraction were increased in isoproterenol-treated and transplanted glands. Isoproterenol increased F-actin polymerization in the apical and lateral regions of the cytoplasm in both control and transplanted glands. Inhibiting PKA activity and/or F-actin formation abolished the isoproterenol-enhanced expression of VAMP-2 and syntaxin-4 in the secretory granule fraction and the isoproterenol-enhanced expression of VAMP-2 in the membrane protein fraction. Taken together, these results indicate that the activation of β-ARs induces secretory granules and cell membrane fusion via the interaction of VAMP-2 and syntaxin-4 in a PKA- and F-actin-dependent manner in human submandibular gland. Up-regulated β-ARs might participate in altering protein secretion in transplanted submandibular gland by promoting the interaction of VAMP-2 with syntaxin-4.

## Introduction

The release of salivary proteins is primarily evoked by sympathetic neurotransmitter action on β-adrenoceptors (β-ARs), which subsequently activates protein kinase A (PKA) [1]. In rat salivary gland, most proteins including amylase and mucin are synthesized and stored in secretory granules. After the activation of β-ARs and PKA, salivary proteins are released via exocytosis, which is achieved by the interaction of vesicle-anchored soluble *N*-ethylmaleimide-sensitive fusion attachment protein receptors (v-SNAREs) with target membrane-anchored SNAREs (t-SNAREs) [2,3]. Amongst these SNAREs, synaptobrevin/vesicle-associated membrane proteins (VAMPs) are v-SNARE family members, whereas syntaxins and synaptosome-associated protein 25 (SNAP-25) homologs are t-SNAREs [4]. In human submandibular gland, VAMP-2, syntaxin-2, syntaxin-4, and SNAP-23, together with cytoskeletal components such as actin, have been detected by immunohistochemistry [5]. However, the exact roles of β-ARs and SNAREs in human submandibular gland protein secretion need to be further studied.

Dry eye syndrome is a common ophthalmologic disorder characterized by reduced or lack of tear production. This abnormality may result in the disruption of the ocular surface, even loss of sight, and interference with quality of life [6]. Autologous transplantation of submandibular gland with insertion of Wharton’s duct into the upper conjunctival fornix is considered a novel strategy to treat severe dry eye syndrome [6–9]. After surgery, salivary-tear from the transplanted gland provides a continuous, endogenous source of ocular lubrication that could substantially improve clinical manifestations and reduce corneal damage. However, the findings of long-term follow-up show that epiphora occurs in more than 40% of patients at 6 months after transplantation, and the total protein content and activities of proteins (such as secretory IgA, lysozyme, and amylase) in salivary-tear are higher than those in normal saliva [10]. Protein secretion from the transplanted gland is altered via an unknown mechanism. More importantly, based on the different composition between salivary-tear and normal tear, the salivary-tear film breakup time is shorter than that of the normal tear. Congestion, inflammation, and metaplasia of the conjunctival epithelium still persist in some patients [10,11]. Therefore, understanding the mechanism of protein secretion from the transplanted gland is critical for regulating transplanted gland secretion.

Notably, the artery and vein of the transplanted submandibular gland are reconstructed by microsurgery, but without nerve anastomosis during the operation. However, a previous study has reported that physical activity and warm temperature increase the secretion of salivary-tear [12]. Catecholamine fluorescence shows scattered adrenergic nerve staining around acini in the transplanted gland [13]. Taken together, these results suggest that the sympathetic system might reappear and participate in the changed protein secretion of the transplanted gland. However, changes in β-ARs and their downstream molecules in the transplanted gland remain unknown.

Therefore, the present study was designed to reveal the role of β-ARs and SNARE-related proteins in the protein secretion of human submandibular gland together with the potential mechanism involved in the protein secretion of transplanted submandibular gland.

## Materials and methods

### Human participants and ethics

Transplanted submandibular gland samples were collected from 12 individuals (aged 26–45 years, 8 males) who underwent partial gland reduction for epiphora within 6–12 months after transplantation (mean: 8.65 months). The submandibular glands from 12 individuals (aged 52–59 years, 10 males) who underwent functional neck dissection for primary oral squamous cell carcinoma without irradiation and chemotherapy served as controls. All control samples were confirmed to be histologically normal. The research protocol was approved by the Institutional Review Board of Peking University Hospital of Stomatology, and prior to tissue collection, all participants signed an informed consent document.

### Submandibular gland tissue preparation

The fresh gland samples were minced into small pieces (0.5 mm^3^), and cultured in Dulbecco’s modified Eagle’s medium (containing 100 U/ml penicillin and 100 μg/ml streptomycin; Gibco, Grand Island, NY, U.S.A.) at 37°C for 30 min in a humidified atmosphere of 5% CO_2_ and 95% air with or without isoproterenol, H89, or cytochalasin B stimulation (all from Sigma–Aldrich, St. Louis, MO, U.S.A.).

### Histological and morphometric analysis

Submandibular gland tissues were fixed in 10% formalin, paraffin-embedded, sectioned at 5 μm, and stained with Hematoxylin-Eosin for morphometric evaluation using the Leica Qwin software (Q550CW, Leica, Mannheim, Germany). Other gland specimens were fixed in 2% paraformaldehyde-1.25% glutaraldehyde. Ultrathin sections were prepared and stained with uranyl acetate and lead citrate and observed by TEM (H-7000 electron microscope; Hitachi, Tokyo, Japan). The area of acinar cells, number of secretory granules, and area of secretory granules were calculated using the imaging software (Q550CW, Leica, Mannheim, Germany) and ImageJ (NIH, Bethesda, MD, U.S.A.). The data were from 9 randomly selected acini in each section from 12 control and 12 transplanted glands.

### Reverse-transcription PCR and quantitative PCR

Total RNA was purified with TRIzol (Invitrogen, Carlsbad, CA, U.S.A.). cDNA was prepared from 2 μg of total RNA with a RevertAid First Strand cDNA Synthesis Kit (Promega, Madison, WI, U.S.A.). The primers were designed according to mRNA sequence of human β1-AR, β2-AR, β3-AR, VAMP-2, syntaxin-2, syntaxin-4, SNAP-23, and GAPDH (Table 1). The band densities were quantitated using a Leica 550IW imaging system (Leica, Manheim, Germany).

**Table 1:** Primer sequences for reverse-transcription PCR

Gene	Upper primer (5′–3′)	Lower primer (5′–3′)
*β1-AR*	GCTCACCAACCTCTTCATC	CAGGGCAATGACACACAG
*β2-AR*	AGCCTGCTGACCAAGAATAAG	GTAGAAGGACACGATGGAAGAG
*β3-AR*	GCCCAATACCGCCAACAC	CCAGCGAAGTCACGAACAC
*VAMP-2*	GACAAGGTCCTGGAGCGAGA	AGGAAGGGGTGTTAAGGACAAC
*Syntaxin-2*	TTGTTGTGGTTGAGAAAGATCA	ACTTCCGAGACAGCACCGAATG
*Syntaxin-4*	CAGATGCTGGACAGTGGGCAAA	CTAGGAGGACGACGGTGATGGA
*SNAP-23*	ATCAAGACCATCACTATGCTGG	TTATGCGTTTAATGTATCCACC
*GAPDH*	GAGTCCACTGGCGTCTTC	GATGATCTTGAGGCTGTTGTC

For quantitative PCR (qPCR), DyNAmo™ ColorFlash SYBR Green qPCR Kit and Thermo PikoReal Real-Time PCR Systems (Thermo Fisher Scientific) were used. The reactions were performed under the conditions of denaturation at 95°C for 7 min, 40 cycles at 95°C for 10 s, and 60°C for 30 s, followed by melting curve analysis to determine the specificity. *C*_t_ values and efficiency were obtained from standard curves with a control sample using serial dilutions of the cDNA. The primer sequences of β1-AR, β2-AR, VAMP-2, syntaxin-4, β-actin, and GAPDH were shown in Table 2. The relative expression ratio of a target gene was presented in comparison with GAPDH using the Δ–Δ method as described previously [14].

**Table 2:** Primer sequences for qPCR

Gene	Upper primer (5′–3′)	Lower primer (5′–3′)
*β1-AR*	CAGACGAGGATTGTGGGCTT	CGGAATCCAAGGTGTAGGGC
*β2-AR*	CCGGTACCAGTGCATCTGAA	GTGATCGCAGTGGATCGCTA
*VAMP-2*	TCCAGCAAGTCCCATACACA	GAAAACCCTCCTCCCCAAT
*Syntaxin-4*	TGCGACATTATCCAACCACT	CAGAAGAAGGCGAGGAAGAA
*β-actin*	TGTTGGCGTACAGGTCTTTG	CTCTTCCAGCCTTCCTTCCT
*GAPDH*	CTTTGGCATTGTGGAAGGGCTC	GCAGGGATGATGTTCTGGGCAG

### Western blot analysis

Protein extracts (20 μg) were separated on SDS/PAGE (12% gel) as described previously [15]. Blocked membranes were incubated with the antibodies against β1-AR (sc-568), β2-AR (sc-569), β3-AR (sc-1473), p-PKA (Thr^198^) (sc-32968), and PKAα (sc-28315) at 1:500 dilution (Santa Cruz Biotechnology, Delaware, CA, U.S.A.); against VAMP-2 (104 211, Synaptic Systems, Göttingen, Germany), at 1:5000 dilution; and against syntaxin-4 (ab77037; Abcam, Hong Kong, China), occludin (33-1500; Invitrogen, Carlsbad, CA, U.S.A.), and GAPDH (TA-08; ZSGB-BIO, Beijing, China) at 1:1000 dilution in TBS-T (20 mM Tris, 137 mM NaCl, 0.1% Tween-20, pH 7.4). Membranes were washed and probed with horseradish peroxidase-conjugated secondary antibodies (1:8000) and developed in an ECL detection system (GE Biosciences, Buckinghamshire, England). Densitometry data were analyzed by Quantity One (Bio–Rad, Hercules, CA).

### Immunofluorescence staining

Gland tissues were sectioned at 5-μm thick and fixed in cold acetone for 15 min. After a washing with PBS (137 mM NaCl, 2.7 mM KCl, 4.3 mM Na_2_HPO_4_, 1.4 mM KH_2_PO_4_, pH 7.4) for three times, tissue slides were stained with Alexa Fluor 488-conjugated Phalloidin (Sigma–Aldrich, St. Louis, MO) at 37°C for 1 h according to the manufacturer’s instructions. For calponin staining, tissue slides were blocked with 20% serum for 30 min, immunostained with anti-calponin (1:100; ZSGB-BIO, Beijing, China) antibody overnight at 4°C, and then incubated with TRITC-conjugated secondary antibodies (1:200; ZSGB-BIO, Beijing, China) for 1 h. After a washing with PBS for three times, nuclei were labeled with DAPI. Images were captured by confocal microscope (Leica TCS SP5, Heidelberg, Germany). The quantitative measurement of F-actin was performed by Leica TCS SP5 (LAS AF) as previously described [16]. The fluorescence intensities of F-actin in nine randomly selected acini in each section of six control and six transplanted glands with or without isoproterenol treatment were averaged. Data are reported as the ratio of F-actin intensity in apical and lateral areas to the total intensity of F-actin in each acinus.

### VAMP-2 co-immunoprecipitation

Gland tissue lysates were incubated with protein A/G-agarose (Santa Cruz Biotechnology, Delaware, CA, U.S.A.) for 1 h at 4°C and then clarified by centrifugation at 15000 ***g*** for 10 min. The supernatants were incubated with anti-VAMP-2 antibody-coated protein A/G overnight at 4°C. After incubation, the immunoprecipitates were washed extensively with lysis buffer and the same amount (20 μg) of protein from control and transplanted glands subjected to SDS/PAGE. Gray values of bands were quantitated and analyzed by ImageJ software (NIH).

### Coomassie Brilliant Blue R-250 staining and in-gel digestion

Gels were stained with Coomassie Brilliant Blue and de-stained to visualize the protein bands. Selected lanes were excised and dried in a Hetovac vacuum centrifuge (HETO, Allerod, Denmark). The dried pieces were rehydrated in 20 ng/μl trypsin and then incubated with NH_4_HCO_3_ overnight. After the pieces were rehydrated, the peptides were eluted in 60% acetonitrile (ACN)/5% trifluoroacetic acid (TFA). The TFA solution containing the proteins was transferred to a polypropylene tube with 60% ACN/5% TFA. A second elution of the peptides was performed with 5% TFA in 60% ACN. The second TFA solution was pooled with the first one. Before mass spectrometry, the volumes of peptide-containing solutions were adjusted to 10 μl by the addition of 0.1% TFA in 60% ACN [16].

### Nano-LC-ESI-MS/MS analysis

The nano-LC-MS/MS experiments were performed using an LTQ Orbitrap Velos Pro mass spectrometer (Thermo Electron, Bremen, Germany). The sample was applied on to an EASY nano-LC system following the protocols of the manufacturer. The general mass spectrometric conditions were: spray voltage, 2.2 kV, no sheath and auxiliary gas flow, ion transfer tube temperature, 250°C, 35% normalized collision energy for MS/MS (MS2). The ion selection threshold was 1000 counts for MS2. An activation q = 0.25 and an activation time of 30 ms were applied in MS2 acquisitions. The mass spectrometer was operated in positive-ion mode and a data-dependent automatic switch was used to switch between MS and MS/MS acquisition modes. For each cycle, one full MS scan in the Orbitrap was followed by 15 MS2 in the LTQ on the ten most intense ions. Selected ions were excluded from further selection for 30 s.

### Database search and data analysis

The LC-MS/MS data were submitted to database searching against the human sequence library in the Uniprot protein sequence database using the Sequest HT algorithm in the Proteome Discoverer 1.4 software package (Thermo Scientific). Trypsin was chosen as the enzyme with a maximum of two missed cleavages allowed. Carbamidomethylation of cysteine was set as static modifications, and oxidation of methionine was set as variable modifications. The MS and MS/MS results were searched with a peptide ion mass tolerance of 10 ppm and a fragment ion mass tolerance of ±0.8 Da. The Percolator-based filter was used to filter results with an FDR <1% [17].

### Secretory granules extraction

Secretory granules were isolated from submandibular gland as described previously [18]. Briefly, the granules were lysed overnight by conventional dialysis against a hypotonic buffer or by placing the resuspended granule pellets in a 65-ml Amicon ultrafiltration chamber and carrying out pressure dialysis [19]. The following morning the lysate was centrifuged at 10000 ***g*** for 15 min to separate the soluble fraction of granule proteins from membranes and other particulate matter.

### Membrane protein extraction

The extraction of membrane protein fractions from submandibular gland was performed using the Membrane Extraction Kit (Applygen, Beijing, China) according to the manufacturer’s instructions.

### Statistical analysis

Data were presented as mean ± S.D. Statistical analysis was performed using an unpaired Student’s *t*test for two groups or one-way ANOVA followed by Bonferroni’s *post hoc* test for multiple groups. *P*<0.05 was considered statistically significant.

## Results

### Histology and ultrastructure of submandibular glands

**Figure 1:**
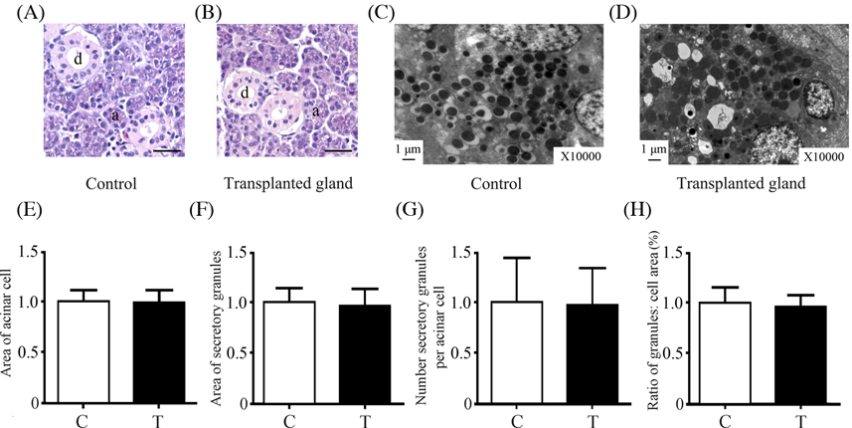
Histology and ultrastructure of control and transplanted glands Representative histological images of control (**A**) and transplanted (**B**) glands; a, acinus; d, duct. Scale bar: 30 μm. Representative ultrastructural images of control (**C**) and transplanted (**D**) glands, the same tissues as in (A,B). Scale bar: 1 μm. The area of acinar cells (**E**), the area of secretory granules (**F**), the number of secretory granules (**G**), and the area ratio of secretory granules: cell (**H**) was analyzed in control and transplanted glands. Abbreviations: C, control gland; T, transplanted gland.

Acini, ducts, and secretory granules in control and transplanted glands were subjected to Hematoxylin-Eosin staining and TEM (Figure 1A–D). In control glands, the acini and secretory granule areas were 654.95 ± 65.59 μm^2^ and 0.91 ± 0.17 μm^2^, respectively, consistent with the results of a previous study [20]. In addition, quantitative analysis showed that the area of acinar cells, the area and number of secretory granules, and the ratio of the secretory granule area to the acinar cell area in transplanted epiphora glands did not differ from those in controls (Figure 1E–H). In addition, we found that there were vacuoles in the cytoplasm of the acinar cells of the transplanted glands, which were not seen in the control glands (Figure 1C,D). Furthermore, vacuole formation was also in continuity with the lumen in transplanted glands, rather than controls (Supplementary Figure S1).

### Expression of β-ARs and PKA in submandibular glands

**Figure 2:**
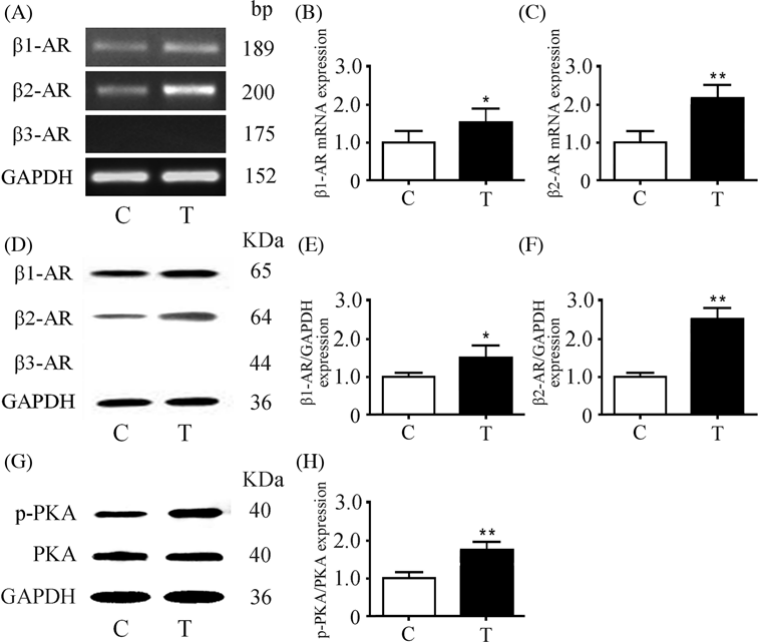
Expressions of β-ARs and PKA in submandibular glands (**A**) mRNA expressions of β1-, β2-, and β3-AR using reverse-transcription PCR (RT-PCR). Relative *β1-* (**B**) and *β2-AR* (**C**) mRNA expressions in control and transplanted glands were determined by qPCR. (**D**) Protein expressions of β1-, β2-, and β3-AR by Western blot analysis with the same tissues as in (A). Quantitative analysis of β1- (**E**) and β2-AR (**F**) protein expressions in control and transplanted glands. (**G**) Expressions of p-PKA and PKA in control and transplanted glands. (**H**) Quantitative analysis of p-PKA and PKA expressions in control and transplanted glands normalized to GAPDH expression. Values are mean ± S.D. from six independent experiments. *P<0.05 and **P<0.01 compared with controls. Abbreviations: C, control gland; T, transplanted gland.

Both *β1-* and *β2-AR* mRNA expressions were observed in control and transplanted glands, whereas *β3-AR* mRNA was not detectable (Figure 2A). Compared with controls, the expression of *β1-AR* and *β2-AR* mRNA in transplanted glands was increased by 58.1 and 117.4%, respectively (Figure 2B,C). The expression of β1- and β2-AR proteins was increased by 49.4 and 155.6%, respectively (Figure 2D–F). PKA is considered a major target of β-ARs in exocytosis [3]. Compared with controls, the expression of p-PKA was increased by 78.6%, whereas the total content of PKA did not change in the transplanted glands (Figure 2G,H).

### Expression of VAMP-2 and syntaxin-4 in submandibular glands

**Figure 3:**
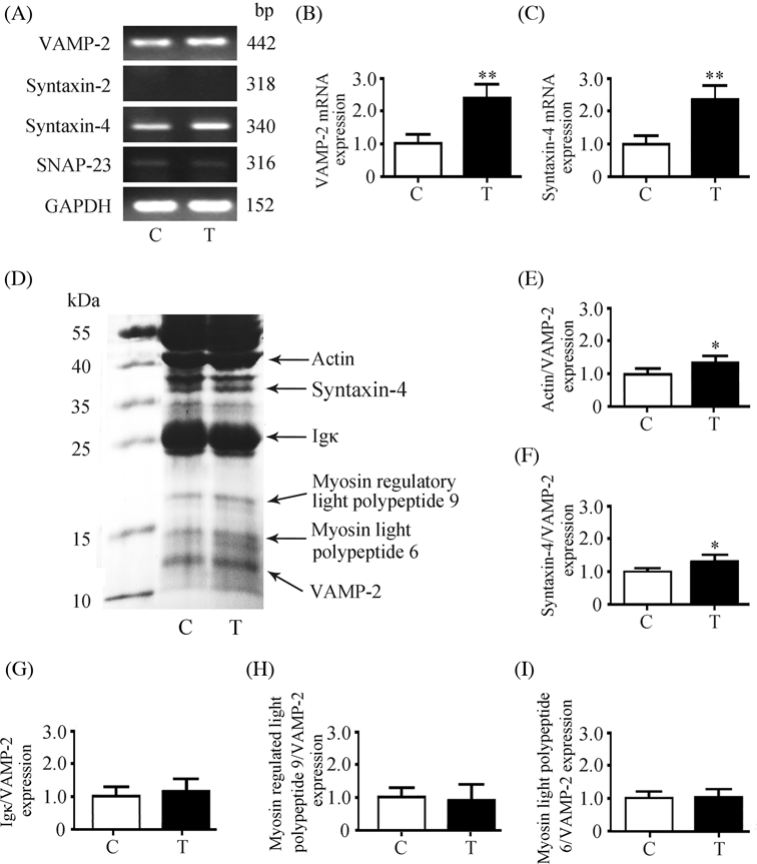
Expressions of SNARE complex in submandibular glands (**A**) Expression of *VAMP-2, syntaxin-2, syntaxin-4*, and *SNAP-23* mRNAs using reverse-transcription PCR (RT-PCR). Relative *VAMP-2* (**B**) and *syntaxin-4* (**C**) mRNA expression in control and transplanted glands was determined by qPCR. (**D**) Representative co-immunoprecipitation results in control and transplanted glands using VAMP-2 antibody. The band intensities of actin, syntaxin-4, Igκ, myosin regulatory light polypeptide 9, and myosin light polypeptide 6 were normalized to VAMP-2 expression. Quantitative analysis of actin (**E**), syntaxin-4 (**F**), Igκ (**G**), myosin regulatory light polypeptide 9 (**H**), and myosin light polypeptide 6 (**I**) interaction with VAMP-2 in control and transplanted glands. Values are the mean ± S.D. from six independent experiments. *P<0.05 and **P<0.01 compared with controls. Abbreviations: C, control gland; T, transplanted gland.

SNARE proteins play important roles in protein secretion [4]. The expressions of *VAMP-2* and *syntaxin-4* mRNA were detected in human submandibular gland, whereas *SNAP-23* mRNA was weakly expressed and *syntaxin-2* mRNA was not detectable (Figure 3A). The expression of *VAMP-2* mRNA was increased by 143.6% and the expression of *syntaxin-4* mRNA was increased by 141.9% in transplanted glands as compared with those in the controls (Figure 3B,C).

### Proteins interacted with VAMP-2 in submandibular glands

VAMP-2, as a v-SNARE molecule, must interact with t-SNARE proteins to mediate membrane fusion and protein secretion [21]. To identify the t-SNARE proteins interacting with VAMP-2 in protein secretion, the control and transplanted gland protein lysates were subjected to immunoprecipitation with an anti-VAMP-2 antibody. After SDS/PAGE, the protein bands were identified by Nano-LC-ESI-MS/MS analysis. As shown in Figure 3D, actin (41.7 kDa), syntaxin-4 (34.2 kDa), Igκ (25.8 kDa), myosin regulatory light polypeptide 9 (20 kDa), myosin light polypeptide 6 (17 kDa), and VAMP-2 (12.7 kDa) were detected. Compared with controls, the actin interacting with VAMP-2 was increased by 29.4% and the syntaxin-4 interacting with VAMP-2 was increased by 25.1% in transplanted glands (Figure 3E,F), whereas the expressions of Igκ, myosin regulatory light polypeptide 9, and myosin light polypeptide 6 interacting with VAMP-2 did not change (Figure 3G–I).

### Expression of VAMP-2 and syntaxin-4 in the secretory granule fraction in isoproterenol-treated and transplanted glands

**Figure 4:**
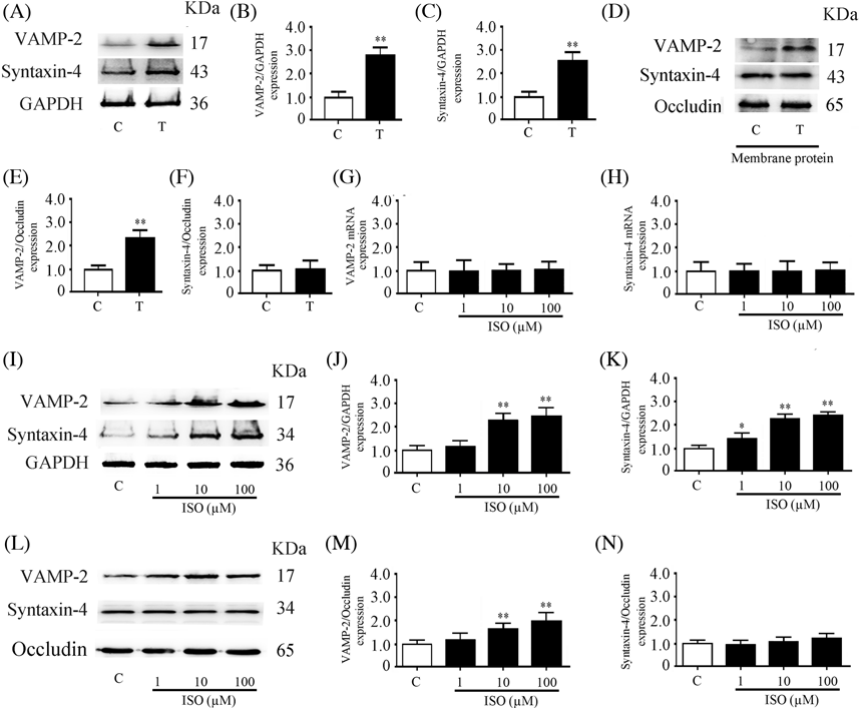
Expression of VAMP-2 and syntaxin-4 in the secretory granule fraction in isoproterenol-treated and transplanted glands (**A**) Western blot analysis of VAMP-2 and syntaxin-4 in the secretory granule fraction of control and transplanted glands. Quantitative analysis of VAMP-2 (**B**) and syntaxin-4 (**C**) expression. (**D**) Western blot analysis of VAMP-2 and syntaxin-4 in the membrane proteins of control and transplanted glands. Quantitative analysis of VAMP-2 (**E**) and syntaxin-4 (**F**) expression. Relative *VAMP-2* (**G**) and *syntaxin-4* (**H**) mRNA expression was determined by qPCR. (**I**) Western blot analysis of VAMP-2 and syntaxin-4 in the secretory granule fraction. Quantitative analysis of VAMP-2 (**J**) and syntaxin-4 (**K**) expression normalized to GAPDH. (**L**) Western blot analysis of VAMP-2 and syntaxin-4 in isolated membrane protein fraction. Quantitative analysis of VAMP-2 (**M**) and syntaxin-4 (**N**) expression normalized to occludin. Values are the mean ± S.D. from six independent experiments. *P<0.05 and **P<0.01 compared with controls. Abbreviations: C, control gland; ISO, control gland with isoproterenol treatment for 30 min T, transplanted gland.

Since the expression and distribution of SNARE proteins play essential roles in protein secretion from the salivary glands [22], we extracted secretory granule and membrane protein fractions. The expression of VAMP-2 and syntaxin-4 in secretory granule fraction from transplanted gland was increased by 187.6 and 157.6%, respectively, compared with those in controls (Figure 4A–C). The content of VAMP-2 in membrane protein fraction was increased by 123.3% in transplanted glands, as compared with controls. However, the expression of syntaxin-4 in membrane protein fraction of the transplanted gland did not change (Figure 4D–F).

To explore the roles of β-ARs in VAMP-2 and syntaxin-4 expression in protein exocytosis of the submandibular gland, freshly isolated control submandibular gland tissues were incubated with 1, 10, and 100 µM isoproterenol, a β-AR agonist, for 30 min. The mRNA expression of VAMP-2 and syntaxin-4 did not change (Figure 4G,H), whereas the contents of VAMP-2 and syntaxin-4 proteins in the secretory granule fraction were significantly increased (Figure 4I–K). We further extracted the membrane protein fraction and found that the VAMP-2 content was increased but that syntaxin-4 expression did not change (Figure 4L–N).

### Isoproterenol increases F-actin polymerization in apical and lateral regions of cytoplasm

**Figure 5:**
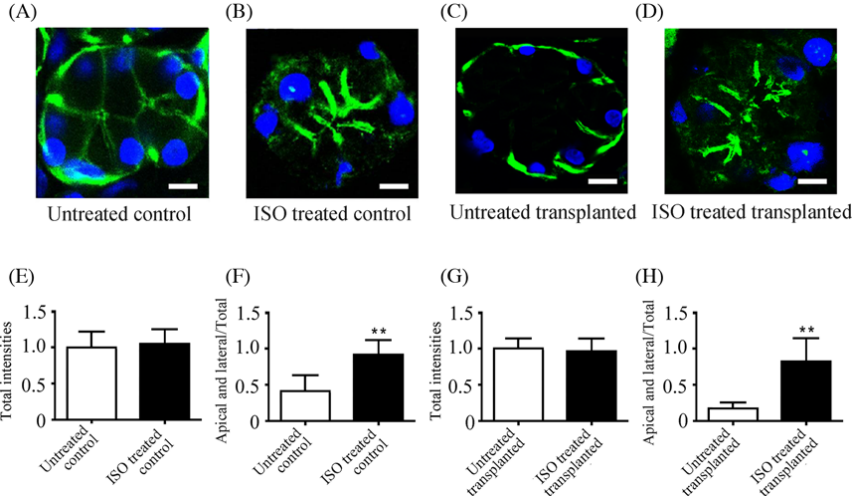
Distribution of F-actin in glands with or without isoproterenol treatment F-actin (green) was stained with Alexa Fluor 488-conjugated Phalloidin. Nuclei were stained with DAPI (blue). Representative images of F-actin in a control gland (**A**), in a normal gland treated with 10 μM isoproterenol for 30 min (**B**), in a transplanted gland (**C**), and in a transplanted gland treated with 10 μM isoproterenol for 30 min (**D**). Scale bars: 8 μm. Quantitative detection of F-actin in nine randomly selected acini in each section from six control and transplanted glands with or without isoproterenol treatment. Both the total intensity (**E**,**G**) and the ratio of F-actin intensity in the apical and lateral regions of the cytoplasm of acinar cells to the total F-actin intensity (**F**,**H**) are shown for control (E,F) and transplanted (G,H) glands with and without isoproterenol treatment. Values are the mean ± S.D. from six independent experiments. **P<0.01 compared with untreated glands. Abbreviation: ISO, isoproterenol.

Actin filaments are involved in salivary gland protein secretion [23]. To identify whether actin filaments could be regulated by β-ARs and examine the role of this response in protein secretion, we observed the distribution of F-actin (a polymerized form of actin) in freshly cultured gland tissues with or without isoproterenol treatment. In untreated control glands, F-actin was uniformly distributed in the apical and lateral regions of the cytoplasm and discontinuously distributed close to the basal region of the cytoplasm (Figure 5A). The co-staining of F-actin and calponin, one of the markers of myoepithelial cells was shown in Supplementary Figure S2. Calponin expression (red) was observed in the periphery of acini, which was consistent with the location of calponin in submandibular gland in a previous study [16]. Although the merged images revealed that F-actin was co-localized with calponin (orange) in myoepithelial cells, the much more positive staining of F-actin (green) was detected in the basal region of acini with negative staining of calponin. Isoproterenol treatment (10 µM for 30 min) increased F-actin staining in the apical and lateral regions of the cytoplasm and decreased F-actin staining in the basal region of the cytoplasm (Figure 5B). In transplanted gland, the intensity of F-actin staining in the apical and lateral regions of the cytoplasm of untreated glands was low, and F-actin was aggregated in the basal region of the cytoplasm (Figure 5C), consistent with our previous study [16]. After isoproterenol stimulation, F-actin was markedly increased in the apical and lateral regions of the cytoplasm and decreased in the basal region of the cytoplasm (Figure 5D). Quantitative analysis further showed that the intensity of total F-actin in the acinar cells of control glands with or without isoproterenol stimulation was not significantly different (Figure 5E), whereas the ratio of F-actin in the apical and lateral regions of cytoplasm to the total F-actin of each acinus was significantly increased by 127% in control glands with isoproterenol stimulation (Figure 5F). In transplanted glands, isoproterenol treatment did not affect the total F-actin intensity (Figure 5G) but significantly increased the ratio of F-actin in the apical and lateral regions of the cytoplasm to the total F-actin of each acinus (Figure 5H). These results suggest that the activation of β-ARs increases F-actin polymerization in the apical and lateral regions of the cytoplasm, which might mediate the movement of secretory granules to apical cytoplasm. In the transplanted gland, the distribution and response of F-actin to β-AR stimulation were changed, which might be involved in the alteration of protein secretion.

### Isoproterenol increases the contents of VAMP-2 and syntaxin-4 in the secretory granule fraction through PKA activation and F-actin integrity

**Figure 6:**
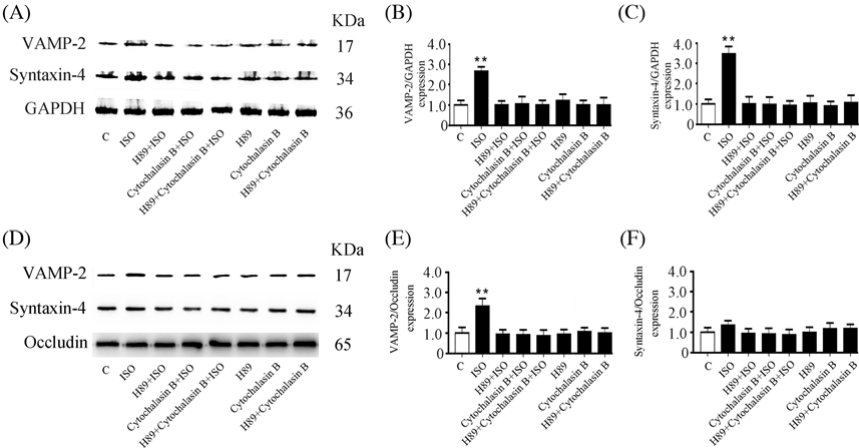
Isoproterenol increases the expression of VAMP-2 and syntaxin-4 in a PKA- and F-actin-dependent manner (**A**) Western blot analysis of VAMP-2 and syntaxin-4 in the secretory granule fraction of glands. Quantitation of VAMP-2 (**B**) and syntaxin-4 (**C**) expression normalized to GAPDH. (**D**) Western blot analysis of VAMP-2 and syntaxin-4 in the membrane protein fraction of glands. Quantitation of VAMP-2 (**E**) and syntaxin-4 (**F**) expression normalized to occludin. C, control gland; ISO, gland treated with 10 μM isoproterenol for 30 min. H89 + ISO, gland pretreated with 10 μM H89 for 30 min and then stimulated with isoproterenol for 30 min. Cytochalasin B + ISO, gland pretreated with 20 μM cytochalasin B for 30 min and then stimulated with isoproterenol for 30 min. H89 + cytochalasin B + ISO, gland pretreated with H89 and cytochalasin B for 30 min and then stimulated with isoproterenol for 30 min. H89, gland treated only with H89. Cytochalasin B, gland treated only with cytochalasin B. H89 + cytochalasin B, gland treated with H89 and cytochalasin B. Values are the mean ± S.D. from six independent experiments. **P<0.01 compared with controls.

We further explored the roles of VAMP-2, syntaxin-4, and actin in saliva secretion induced by β-AR activation. VAMP-2 expression was increased by 171.3% and syntaxin-4 expression increased by 243.1% in the secretory granule fraction compared with untreated gland (Figure 6A–C). Inhibiting the activity of PKA with H89 and/or inhibiting F-actin formation with cytochalasin B abolished the isoproterenol-enhanced expression of VAMP-2 and syntaxin-4 in the secretory granule fraction, and similar results were observed when H89 and cytochalasin B were administered together. H89, or cytochalasin B alone, or in combination had no effect on the expression of VAMP-2 or syntaxin-4 (Figure 6A–C). Additionally, isoproterenol increased the content of VAMP-2, but not syntaxin-4, in the membrane protein fraction of the gland, which was abolished by H89 and/or cytochalasin B pretreatment (Figure 6D–F). These results suggest that the β-ARs activation-induced VAMP-2 interaction with syntaxin-4 and actin is dependent on PKA activation and F-actin integrity.

## Discussion

The present study demonstrated that the activation of β-ARs increases VAMP-2 and syntaxin-4 expression in the secretory granule fraction and VAMP-2 expression in the membrane protein fraction, which was dependent on PKA activation and F-actin formation in the apical and lateral regions of the cytoplasm. In transplanted epiphora gland, β-AR signaling pathway was up-regulated as characterized by increased β1-AR and β2-AR expression and PKA phosphorylation. The content of VAMP-2 and syntaxin-4 in the secretory granule fraction and the content of VAMP-2 in the membrane protein fraction were increased. These results suggested that the up-regulated β-ARs might participate in the altered protein secretions in the transplanted gland. Taken together, the present study revealed a novel intracellular mechanism involved in β-AR-modulated protein secretion in physiological and pathological conditions of human submandibular gland.

The activation of β-ARs by norepinephrine released from adrenergic fibers mediates protein secretion in the salivary gland [24,25]. The absence of norepinephrine and down-regulation of β-ARs contribute to hyposecretion in the early phase of rabbit submandibular gland transplantation, and isoproterenol increases fluid secretion and amylase expression by increasing β-AR expression [26]. However, the role of β-ARs in protein secretion in human gland transplantation with changed protein secretion remains unknown. Here, we found that although the morphology of acini and ducts, especially the secretory granules in the transplanted human gland, was comparable with that of controls, the β1-AR and β2-AR expression and PKA phosphorylation were increased in the transplanted gland. These results suggest that the up-regulation of the β-AR signaling pathway might be involved in altering the secretions of the transplanted gland.

In addition, we found the vacuoles in the acinar cytoplasm of the transplanted gland rather than control gland. Previous studies reported that carbachol, an agonist of muscarinic cholinergic receptor (mAChR), induces vacuoles formation in the parotid gland cells via increased intercellular Ca^2+^ ([Ca^2+^]_i_) [27,28]. We have also demonstrated that M1- and M3-mAChR is up-regulated and carbachol-induced [Ca^2+^]_i_ mobilization is increased in the transplanted gland obtained from epiphora patients [29]. Here, β-ARs were increased and vacuoles’ formation was in continuity with the lumen in the transplanted gland with the changed protein secretion. These results suggest that vacuoles’ formation might involve in the fluid and protein hypersecretion of the transplanted gland. The role of β-ARs in the changed protein secretion of the transplanted gland need to be further studied.

Following β-AR and PKA activation, the proteins are released from the secretory granules via exocytosis [2]. The fusion of secretory organelles with cell membranes is a fundamental exocytosis event achieved by the SNARE complex [4]. As a v-SNARE molecule, the cleavage of VAMP-2 partially but significantly inhibits amylase release, suggesting that VAMP-2 plays a key role in protein secretion by exocrine cells [30]. A previous study reported that VAMP-2 and VAMP-3 interact with syntaxins and participate in protein secretion in rat parotid acinar cells [31]. However, t-SNAREs co-precipitated with VAMP-2 were not detectable in another study [32]. Here, we found that syntaxin-4 immunoprecipitated with VAMP-2 in both control and transplanted glands, which indicated that syntaxin-4 is a candidate t-SNARE that interacts with VAMP-2 in human submandibular gland.

In labial salivary glands from the patients with Sjögren’s syndrome, syntaxin-4 and VAMP-8 expression were decreased, whereas syntaxin-3 expression was increased [22]. Moreover, in these patients, aberrant localization of SNARE proteins translocated from the apical to the basal region of acinar cells is considered to be linked to the ectopic mucin secretion [22]. These studies indicate that the expression and distribution of SNARE proteins play essential roles in the protein secretion of salivary gland. Here, we found that the contents of VAMP-2 and syntaxin-4 in the secretory granule fraction and the content of VAMP-2 in the membrane protein fraction were increased in the transplanted gland, suggesting that the redistribution of VAMP-2 and syntaxin-4 might be involved in the changed protein secretion of transplanted gland.

To further confirm the role of VAMP-2 and syntaxin-4 in the protein secretion induced by β-ARs activation, we used isoproterenol to stimulate human submandibular gland, and found that the activation of β-ARs increased the content of VAMP-2 and syntaxin-4 in the secretory granule fraction and the content of VAMP-2 in the membrane protein fraction. These results suggest that β-AR activation promotes secretory granules fusing with the plasma membrane and SNARE complex formation. In transplanted glands, the interaction of VAMP-2 and syntaxin-4, and the VAMP-2 content in the membrane protein fraction were increased, which might be involved in changed protein secretion.

Cytoskeletal components, such as actin filaments, are involved in salivary gland protein secretion [23]. Several studies have reported that F-actin located under the plasma membrane prevents secretory granules from reaching their exocytotic destination. In human parotid and submandibular gland, secretory granules are separated from the luminal membrane by F-actin [33]. After stimulation, the actin cytoskeleton is rearranged and disassembled, consequently inducing secretory granules to reach their sites of exocytosis [34]. In contrast, another study indicated that F-actin plays a positive role in gland secretion by regulating the formation of vesicles and their movement into the cell membrane [35]. Similarly, the depolymerization of F-actin inhibits exocytosis and prevents amylase release in rat submandibular gland [36,37]. Here, we found that F-actin was aggregated in the basal region in the transplanted glands. Isoproterenol induced the polymerization of F-actin in the apical and lateral regions of the cytoplasm and the depolymerization of F-actin in the basal region of the cytoplasm in both control and transplanted glands. These results suggested that the activation of β-ARs induced the polymerization of F-actin in the apical and lateral regions, which might facilitate the movement of secretory granules to the apical membrane.

To further confirm the role of PKA and F-actin in the protein secretion from the submandibular gland, we used the inhibitors of PKA activity and F-actin formation. Isoproterenol increased VAMP-2 and syntaxin-4 expression in secretory granules, and VAMP-2 expression in the membrane protein fraction was abolished by H89 or cytochalasin B, suggesting that PKA activation and F-actin integrity were required for β-AR-enhanced secretory granule movement and SNARE complex formation.

In summary, we provided evidence that the activation of β-ARs induces secretory granules and cell membrane fusion via the interaction of VAMP-2 and syntaxin-4 in a PKA- and F-actin-dependent manner in human submandibular gland. The up-regulation of β-ARs might be involved in the alteration of protein secretion in transplanted submandibular gland. These findings will enhance the current understanding of the protein secretory mechanism in the submandibular gland and help to find the potential therapeutic targets to regulate the secretion of the transplanted gland.

## Clinical perspectives

- Autologous submandibular gland transplantation is an effective treatment for severe dry eye syndrome. However, the protein secretion from the transplanted gland is altered through an unknown mechanism. Here, we aimed to explore the role of β-ARs and their downstream molecules in the transplanted gland and reveal the mechanism of β-ARs in protein secretion.
- The present findings demonstrated that β-ARs activation induces the interaction of VAMP-2 and syntaxin-4 in secretory granules through a PKA and F-actin-dependent manner in human submandibular gland. In the transplanted gland, the up-regulated β-AR signaling pathway might participate in changed protein secretion via promoting the interaction of VAMP-2 with syntaxin-4.
- The present study will deepen the current understanding of the protein secretory mechanism in human submandibular gland and help to find a potential target to regulate the secretion of transplanted gland to mimic normal tears and stabilize the ocular surface.

## Supporting information

**Supplementary Figure 1:** Representative ultrastructural images of control and transplanted glands. Vacuoles are in continuity with the lumen in the transplanted glands. Scale bar, 1 μm.

**Supplementary Figure 2:** Co-staining of F-actin (green) and calponin (red) in human submandibular gland. Three human submandibular gland samples were collected and stained with Alexa Fluor 488-conjugated phalloidin and anti-calponin antibody. F-actin positive staining with calponin expression was shown orange in the merged picture. Scale bars, 10 μm.

## Abbreviations

ACN: acetonitrile
β-AR: β-adrenoceptor
[Ca^2+^]_i_: intercellular Ca^2+^
Ct: cycle threshold
FDR: false-discovery rate
GAPDH: glyceraldehyde-3-phoshate dehydrogenase
LTQ: linear trap quadropole
mAChR: muscarinic cholinergic receptor
MS2: MS/MS
PKA: protein kinase A
qPCR: quantitative PCR
SNAP: synaptosome-associated protein
SNARE: soluble *N*-ethylmaleimide-sensitive fusion attachment protein receptor
t-SNARE: target membrane-anchored SNAREs
TFA: trifluoroacetic acid
TRITC: tetramethylrhodamine
VAMP-2: synaptobrevin/vesicle-associated membrane protein
v-SNARE: vesicle-anchored SNARE
