# Supplementary material for: β-adrenoceptor activation increased VAMP-2 and syntaxin-4 in secretory granules are involved in protein secretion of submandibular gland through the PKA/F-actin pathway
Source: Biosci Rep. 2018 Feb 13;38(1):BSR20171142. doi: 10.1042/BSR20171142 (PMC5809613; doi:10.1042/BSR20171142)
Supplement: Supplementary file 1 [file bsr20171142_Supp1.pdf]

Supplementary Figure 1. Representative ultrastructural images of control and transplanted glands. Vacuoles are in continuity with the lumen in the transplanted glands. Scale bar, 1 µm.

Supplementary Figure 2. Co-staining of F-actin (green) and calponin (red) in human submandibular gland. Three human submandibular gland samples were collected and stained with Alexa Fluor 488-conjugated phalloidin and anti-calponin antibody. F-actin positive staining with calponin expression was shown orange in the merged picture. Scale bars, 10 µm.

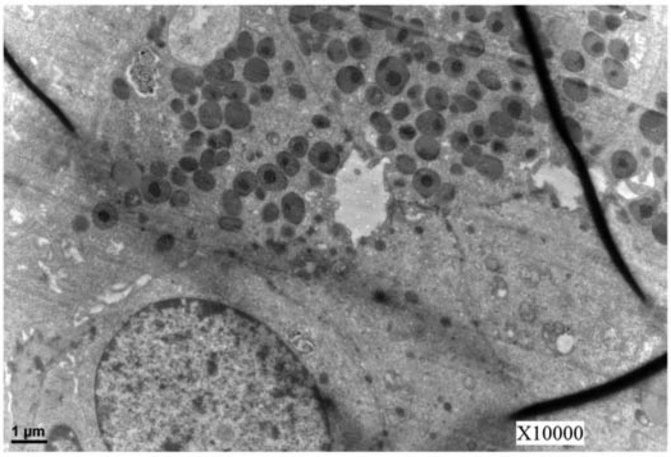

Control gland

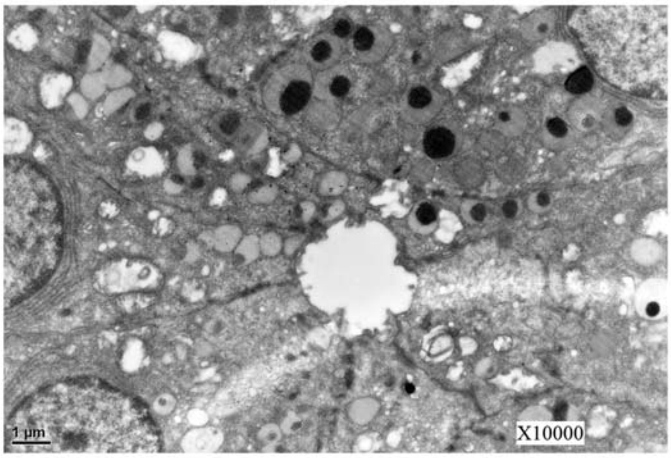

Transplanted gland

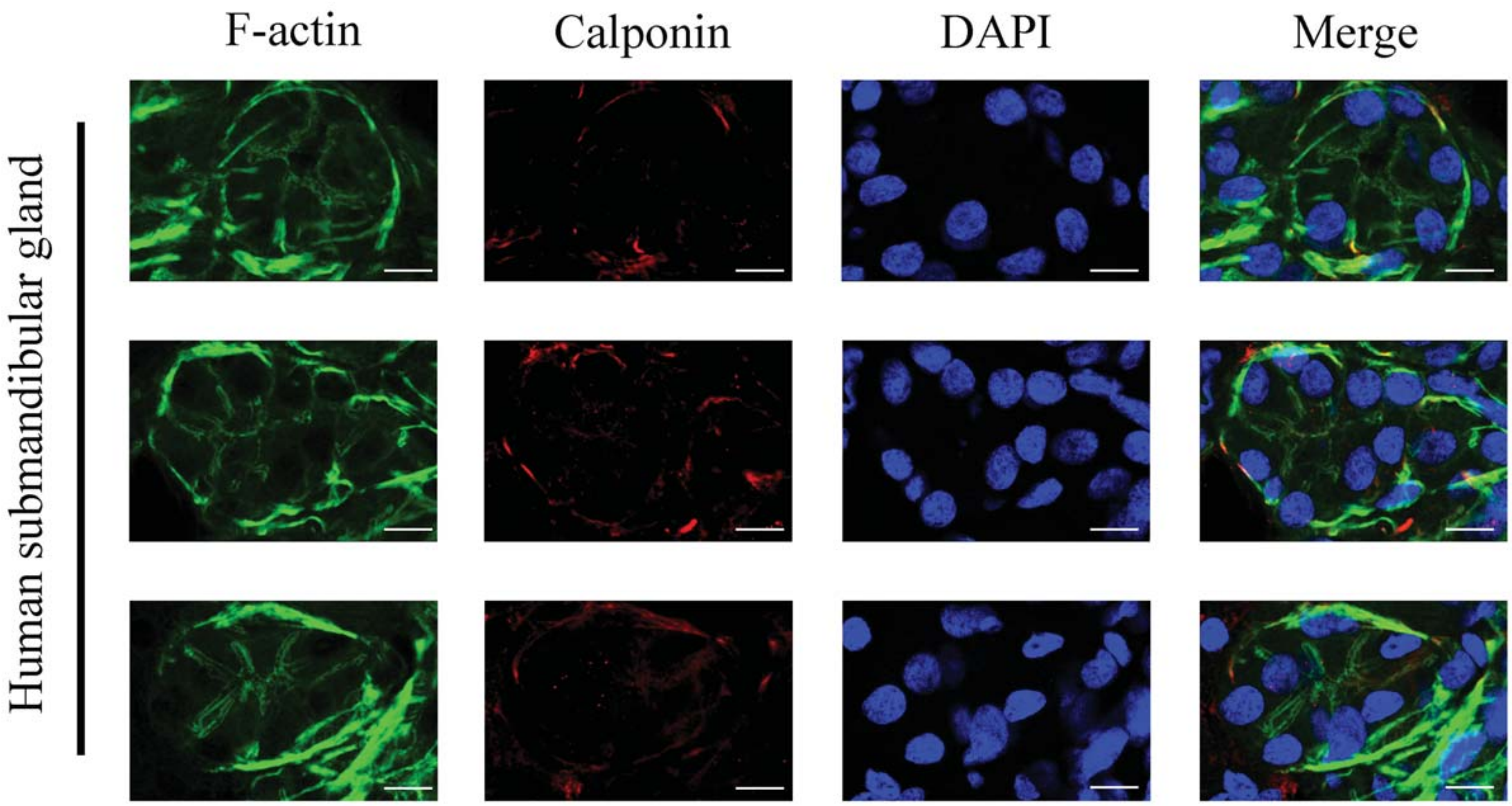

F-actin
Calponin
DAPI
Merge
Human submandibular gland
